# Editorial: Action and Mechanism of Herbal Glycans

**DOI:** 10.3389/fphar.2022.883055

**Published:** 2022-03-24

**Authors:** Shaoping Li, Manuel A. Coimbra, Jing Zhao

**Affiliations:** ^1^ State Key Laboratory of Quality Research in Chinese Medicine, University of Macau, Macao, Macao SAR, China; ^2^ Joint Laboratory of Herbal Glycoengineering and Testing, University of Macau, Macao, Macao SAR, China; ^3^ Department of Chemistry, University of Aveiro, Aveiro, Portugal

**Keywords:** action, mechanism, herb, glycan, intestinal microbial flora

Extensive evidence shows that natural glycans from medicinal plants, fungi and marine organisms have a variety of biological activities with beneficial effects to human body. Discovering target molecules, such as glycan receptors, growth or angiogenesis factors and enzymes, is a key step to track the signaling pathway involved in the succession of glycan effects and understand the pathological mechanisms. Actually, glycans are still the “sleeping giant” of research in herbal medicine, and how they contribute to phytotherapeutic effects are still not clear. Generally, the therapeutic efficacy of a drug mainly depends on its concentration at the site of action. Therefore, how polysaccharides are absorbed and distributed to the site of action is always questionable, as well as their mechanisms of action. Technological advances in glycobiology and glycochemistry are paving the way for a new era in developing sweet solutions to sticky situations.

In this Research Topic of *Action and Mechanism of Herbal Glycans*, a perspective article by Xie et al. proposes polysaccharides as potential therapeutic agents of epilepsy. Besides regulation of inflammatory factors, neurotransmitters, ion channels, and antioxidant reactions, the beneficial effects of polysaccharides treating epilepsy may mainly derive from their targeting on intestinal microbial flora, considered as “intestinal brain organ” or “adult’s second brain”, through “brain gut axis”. Indeed, *Astragalus* (*Astragalus membranaceus* (Fisch.) Ege. var. *mongholicus* (Ege.) Hsiao) polysaccharides (APS) could ameliorate diet-induced cholesterol gallstone formation in mice, and the protective effect of APS, at least partially, results from modulation of gut microbiota (Zhuang et al.). APS seems to improve the diversity of the gut microbiota and increase the relative abundance of the Bacteroidetes phylum, suggesting that APS might be a potential strategy for the prevention of cholesterol gallstone disease. An inulin (CPPF) from Codonopsis Radix (*Codonopsis pilosula* (Franch.) Nannf.) (Zou et al.) was shown to have restorative effects on intestinal mucosal immunity, anti-inflammatory activity and gut microbiota of immunosuppressed mice. As a potential prebiotic substance, a gut microbiota restorative effect was presented by mainly modulating the relative abundance of *Eubacteriales*, including *Oscillibacter*, unidentified *Ruminococcus* and Lachnospiraceae. All results indicated that CPPF was a medicinal prebiotic with enhancing mucosal immune, anti-inflammatory and microbiota modulatory activities.

Cardiovascular protective effects of plant polysaccharides (Dong et al.) were reviewed. These effects derive from anti-oxidative stress, restoring the metabolism of biological macromolecules, regulating the apoptosis cascade to reduce cell death, and inhibiting inflammatory signal pathways, which is beneficial for developing more effective drugs with low side effects for management of cardiovascular diseases. Burdock (*Arctium lappa* L.) fructooligosaccharide (Ding et al.), a water-soluble inulin-type oligosaccharide, was shown potential to protect rat renal tubular epithelial cells (NRK-52E cells) against the reduced cell viability and significantly increased apoptosis rate induced by high glucose, through inhibiting apoptosis and oxidative stress through the Nrf2/HO-1 signaling pathway. Pomegranate peel (*Punica granatum* L.) polysaccharides (Chen et al.) was shown to have potential to ameliorate the symptoms of psoriasis through inhibition of the inflammatory cytokines by suppressing NF-κB and STAT3 signaling pathways and improving skin barrier protection via enhancing aquaporin-3 and filaggrin. Theoretical and experimental evidence has been provided for clinical application of pomegranate peel polysaccharides against psoriasis.

One of the major pharmacological activities of polysaccharides is immunomodulation. Structural characterization and immunomodulatory activity of a novel polysaccharide (LHPW) from Lycopi Herba (*Lycopus lucidus* Turcz. var. *hirtus* Regel) (Zhang et al.) were determined. LHPW was mainly composed of galactose, glucose, fructose and arabinose. It was shown to be able to activate macrophage RAW264.7 and promote splenocyte proliferation. Ginseng (*Panax ginseng* C. A. Mey.) polysaccharides (WGP) showed to significantly increase mRNA and protein levels of complement component 4, one of the core components of the complement system in human hepatocytes (Liu et al.). E-box1 and Sp1 regions play key roles in WGP-induced component 4 transcription. The results provide a new explanation for the intrinsic mechanism by which ginseng boosts human immune capacity. Glycoproteins from *Isodon japonicus* var. *glaucocalyx* (Maxim.) H.W.Li. (Ren et al.) also seems to regulate macrophage polarization and alleviate lipopolysaccharide-induced acute lung injury in mice via TLR4/NF-κB pathway, providing evidence that supports the traditional application of *Isodon japonicus* var. *glaucocalyx* (Maxim.) H.W.Li. in inflammation-linked diseases. A bee pollen polysaccharide from *Rosa rugosa* Thunb (Yang et al.) was reported to promote pancreatic β-Cell proliferation and insulin secretion. In alloxan-induced diabetic mice, oral administration of the acidic fraction of bee pollen polysaccharide effectively decreased the blood glucose, drink intake and urine. It directly stimulated phosphorylation of p38, ERK and AKT to maintain the islet function and mass. In addition, Mori Fructus (*Marus alba* L.) polysaccharides (Bian et al.) was shown to attenuate alcohol-induced liver damage by regulating fatty acid synthesis, degradation and glycerophospholipid metabolism in mice, and effects of different molecular weight polysaccharides from *Dendrobium officinale* Kimura & Migo (Tao et al.) on human colorectal cancer and transcriptome were also reported.

**FIGURE 1:**
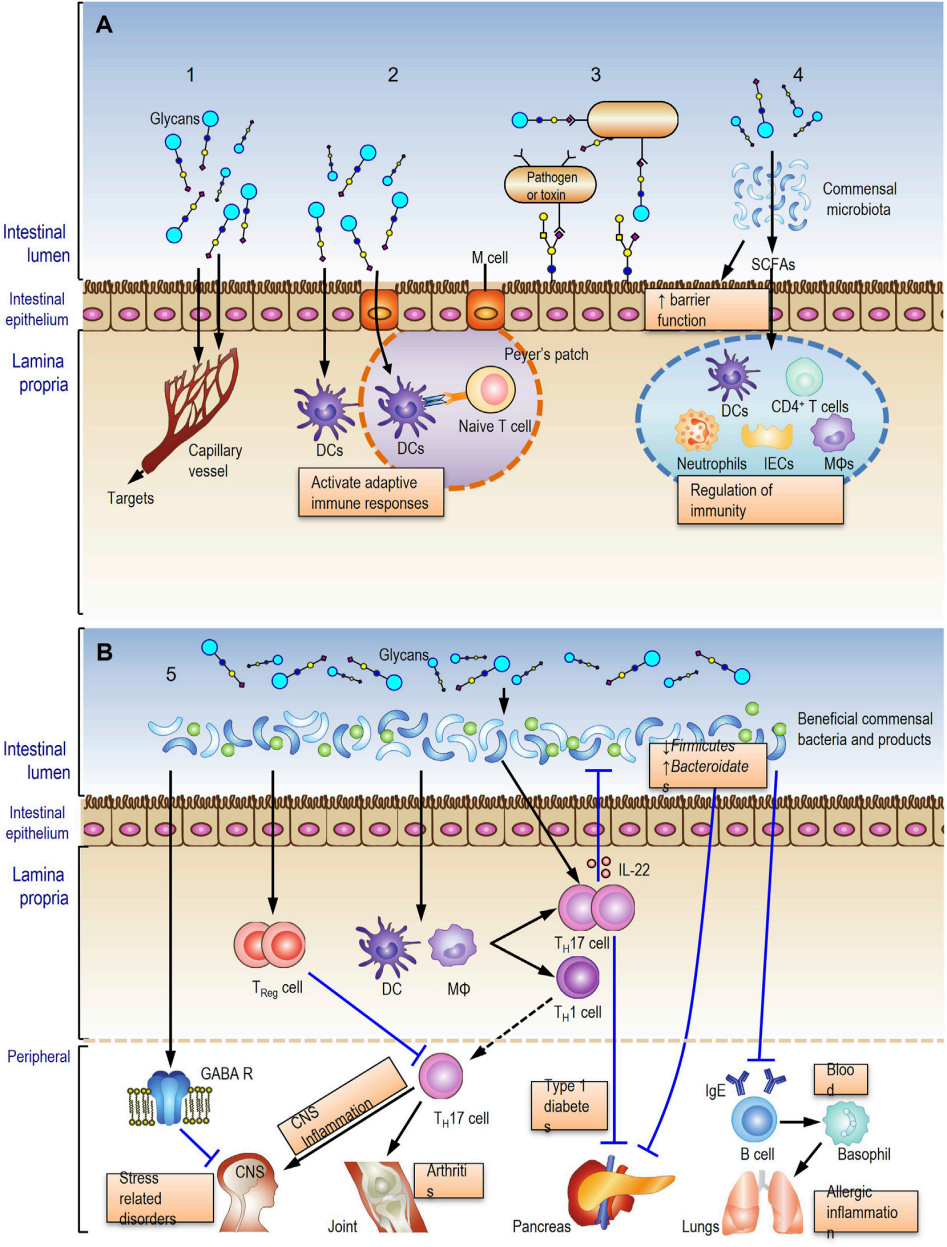
Five potential action modes of oral herbal glycans. Modes 1-4 **(A)** and mode 5 **(B)** described in fulltext.

In summary, the complex relationship between glycans, the gut microbiota and host health are currently considered to comprehensively underpin the multiple activities of herbal glycans. We hypothesized five action modes for oral herbal polysaccharides (Figure 1) based on current evidence, which will be helpful to comprehensively understand the action and mechanism of herbal glycans.

1. Few polysaccharides or their digested oligosaccharide fragments may be absorbed into bloodstream to act on the targets (Figure 1A, mode 1).
2. Polysaccharides or their digested fragments initiate intestinal adaptive immune responses in Peyer’s patches or in mesenteric lymph nodes (Figure 1A, mode 2).
3. Glycans in glycosylated molecules recognize and bind to the pathogen resulting in limited adhesion and a reduced risk of infection (Figure 1A, mode 3).
4. Glycans are metabolized by the gut microbiota into short-chain fatty acids (SCFAs) to modulate immune response (Figure 1A, mode 4).
5. Glycans interact with the gut microbiota and induce beneficial commensal bacteria growth to improve the host health (Figure 1B).

Last but not least, we appreciate all authors, reviewers and guest editors for their contribution. We also would like to take the opportunity of this Research Topic publication to celebrate 70th birthday of Professor Hui Ji, Chinese Pharmaceutical University, China.

